# Growth suppression by altered (p)ppGpp levels results from non-optimal resource allocation in *Escherichia coli*

**DOI:** 10.1093/nar/gkz211

**Published:** 2019-03-27

**Authors:** Manlu Zhu, Xiongfeng Dai

**Affiliations:** School of life sciences, Central China Normal University, Wuhan, Hubei Province, China

## Abstract

Understanding how bacteria coordinate gene expression with biomass growth to adapt to various stress conditions remains a grand challenge in biology. Stress response is often associated with dramatic accumulation of cellular guanosine tetra- or penta-phosphate (p)ppGpp (also known as ‘magic spot’), which is a key second messenger participating in regulating various biochemical and physiological processes of bacteria. Despite of the extensive studies on the mechanism of gene regulation by (p)ppGpp during stringent response, the connection between (p)ppGpp and bacterial steady-state exponential growth remains elusive. Here, we establish a versatile genetic approach to systematically perturb the (p)ppGpp level of *Escherichia coli* through titrating either the single-function (p)ppGpp synthetase or the singe-function (p)ppGpp hydrolase and quantitatively characterize cell growth and gene expression. Strikingly, increased and decreased (p)ppGpp levels both cause remarkable growth suppression of *E. coli*. From a coarse-grained insight, we demonstrate that increased (p)ppGpp levels limit ribosome synthesis while decreased (p)ppGpp levels limit the expression of metabolic proteins, both resulting in non-optimal resource allocation. Our study reveals a profound role of (p)ppGpp in regulating bacterial growth through governing global resource allocation. Moreover, we highlight the Mesh1 (p)ppGpp hydrolase from *Drosophila melanogaster* as a powerful genetic tool for interrogating bacterial (p)ppGpp physiology.

## INTRODUCTION

Microbial cells must constantly adapt their growth to rapidly changing environments. The change of growth rate is always interconnected to a remarkable alteration in the global gene expression pattern (1–8). One of the best-characterized examples is the coordination between gene expression and cell growth of *Escherichia coli* cells growing under different nutrient conditions (1–3,9,10). In rich medium where the nutrient quality is high, *E. coli* cells allocate a major proteome fraction into ribosome proteins and affiliated proteins (e.g., EF-Tu, EF-G) in order to achieve a high protein synthesis rate and fast growth (2,9,11,12). However, in poor medium where the nutrient quality is low, *E. coli* cells must allocate a major proteome fraction to metabolic proteins for nutrient uptake and metabolism at the expenses of ribosome abundance, resulting in slow growth (1,2,13). Although the gene expression patterns of bacteria in response to various growth conditions have been extensively studied (1,14–17), much less is known regarding the signaling systems employed by bacteria to manipulate the global gene expression pattern. Do there exist simple and predictable global signaling strategies for bacteria to govern global resource allocation?

The alarmone guanosine tetra- or penta-phosphate (p)ppGpp (shortened as ppGpp below), known as ‘magic spot’, is one of the three key secondary messengers in bacteria (the other two are cyclic AMP and c-di-GMP) (18). Under stressful conditions (e.g. amino acid deprivation, iron limitation, osmotic shock), a drastic accumulation of ppGpp is observed inside the bacteria cells, known collectively as ‘stringent response’ (18,19). The ‘magic’ and unusual significance of ppGpp signaling is manifested by its involvement in regulating various biochemical and physiological processes of bacteria. The alarmone ppGpp affects an incredibly diverse set of biochemical processes including DNA replication initiation/elongation, RNA synthesis, ribosome synthesis/maturation and translation initiation/elongation process (20–23). Moreover, ppGpp was found to regulate many physiological events of bacteria such as persistence, survival, virulence, pathogenesis, biofilm formation, motility and competence (18,19,21).

To date, much progress has been made in understanding the physiological functions of ppGpp during stringent response. During amino acid starvation, the drastic accumulation of ppGpp effectively shuts down the synthesis of stable RNA (rRNA and tRNA) and reprograms RNA polymerase to active the expression of proteins involved in amino acid biosynthesis (24–26). Recent biochemical and structural studies have further revealed the molecular details of ppGpp in regulating transcriptional initiation process through direct binding to RNAP (27,28). The stringent response reflects the adaptation of bacterial cells to adverse conditions that cause abrupt growth arrest like amino acid starvation. In the life cycle of bacteria, a key stage is exponential growth (29), which is an important parameter reflecting the competitiveness of bacterial populations to occupy nutrient sources. However, the connection between ppGpp and the exponential growth remains a fundamental question. It is known qualitatively that high ppGpp levels efficiently suppress bacterial growth (21,30,31). On the other hand, low ppGpp levels seem to be adverse for biomass growth as well, as shown by the inability of ppGpp-null strain to grow in minimal medium (21,32,33), suggesting the importance of maintain an optimal ppGpp level for cell growth. It is hypothesized that the lack in the expression of proteins involved in amino acid biosynthesis may underlie the failure of ppGpp-null strain to grow in minimal medium (25). However, it is difficult to test related hypotheses due to the lack of a systematic approach to lower the ppGpp pools in minimal medium. Therefore, the details of the interconnection among ppGpp, gene expression and steady-state cell growth remain exclusive. One severe issue in the field of ppGpp physiology is the technical limitation of the frequently used ppGpp-null strain, Δ*relA*Δ*spoT* mutant (33). This mutant is amino acid auxotrophic and can only grow in rich medium containing various amino acids where the ppGpp level of wild type cells is already very low (11,32,33). Moreover, it obtains suppressor mutations easily and suffers viability loss during stationary phase when growing in rich medium (21,34). The technical limitation of ppGpp-null strain has led to some significant controversies regarding the ppGpp function under steady-state growth. For example, a recent study has shown that the ppGpp-null strain exhibits loss of control in ribosome synthesis, supporting ppGpp as the major source of growth control under different nutrient conditions (32). However, earlier findings have reported contradictory results, showing that ppGpp-null strain still exhibits remarkable growth-dependent control of ribosome synthesis (35,36). Therefore, our understanding of the connection between ppGpp and steady-state exponential growth has been severely hindered by the technical limitation.

To investigate the detailed connection of ppGpp to gene expression and cell growth, a straightforward way is to systematically increase or decrease the ppGpp levels of *E. coli*. The wild type *E. coli* cells growing in minimal medium have remarkably higher ppGpp pools than their counterparts growing in rich medium (37), making it important to study the effect of decreased ppGpp levels on cell growth and gene expression in minimal medium. The ppGpp-null strain cannot grow in minimal medium due to the complete absence of ppGpp pools; therefore, an approach to gradually reduce the ppGpp level might address this limitation. In this study, we systematically increase or reduce the ppGpp levels of *E. coli* through titrating the expression of single–function RelA ppGpp synthetase or the single-function Mesh1 ppGpp hydrolase from *Drosophila melanogaster*, respectively. We further quantitatively investigate the effect of altered ppGpp levels on growth rate and gene expression. Our finding reveals a profound role of ppGpp in regulating cell growth through governing global resource allocation.

## MATERIALS AND METHODS

### Strains

All strains used in this study were derivatives of *E. coli* wild type K-12 NCM3722 strain (38,39). The *relA*^+^ gene (containing the N-terminal 455 residues of native *relA* gene) or the inactive *relA’* gene (containing the N-terminal 331 residues of native *relA* gene) was under the control of IPTG-inducible *Ptac* promoter in the pLAS14 vector containing the *PlacIq-lacI* cassette, as described in Xiao *et al.* and Svitil *et al.* (30,33). The coding sequence of Mesh1 protein (40) (optimized based on the codon bias of *E. coli*) was *de novo* synthesized by Tsingke Biotechno. The *mesh1* gene and *spoT E319Q* gene were PCR amplified using the Hieff Canace Gold PCR master mix (Yeasen Biotech, Shanghai, China) and inserted into the EcoRI/HindIII sites of the pLAS14 vector (30), being thus also driven by the IPTG-inducible *Ptac* promoter. The related overexpression vector of RelA^+^, Mesh1 or SpoT E319Q was then transformed into wild type NCM3722 strain and NQ122 strain (*PlacZ*::*km*-*P_Ltet-O1_*-*lacZ*) for measuring RNA/protein ratio or *PLtetO-lacZ* activity, respectively. The Mesh1 or SpoT E319Q vector was transformed into NQ373 (*PlacZ*:*:km-rrnBT*-P*glpF, ΔlacI, ΔlacY, ΔglpR*), NQ554 (*PlacZ::km-rrnBT-PfucP, ΔlacI, ΔlacY*), NQ980 (*PlacZ::km-rrnBT-PlysC, ΔlacI, ΔlacY*) and NQ481 strain (*PlacZ::km-rrnBT-PthrA*) (13) for measuring *PglpFK-lacZ, PfucP-lacZ, PlysC-lacZ* and *PthrA-lacZ* activity, respectively. For constructing the *PsdhC-lacZ* reporter strain, the entire promoter region and upstream sequence of the *sdhCDAB*-*sucABCD* operon together with the first seven codons of the *sdhC* gene (–400 to +21, relative to the translational start site of *sdhC* gene) were PCR amplified and inserted into the HindIII/BamHI site of the low copy RK2-derived vector, pGD926 (41), being fused in frame with the eighth codon of the *lacZ* gene. This procedure generates pGD-PsdhC vector. The pGD-*PsdhC* vector was further transformed into a Mesh1 vector-harboring *lacZ* deficient strain (*ΔlacI, ΔlacZYA*) for measuring *PsdhC-lacZ* activity. All plasmids used in this study were extracted by the DNA plasmid extraction kit of Tsingke Biotech, China.

The NCM3722 Δ*relA*::*kan* strain was made by transferring the *relA::kan* allele from BW25113 Δ*relA*::*kan* strain in Keio collection into the wild type NCM3722 strain through P1 transduction. The NCM3722 Δ*relA*Δ*spoT* strain was further obtained through transferring the *spoT207*::*cat* cassette from strain CF1693 (32) to NCM3722 Δ*relA*::*kan* strain using P1 transduction.

### Medium

Growth media used in this study included LB rich medium, MOPS Rich defined medium (RDM) (9) and MOPS-buffered minimal medium. LB medium (Coolaber, Beijing) contained 10 g/l tryptone, 5 g/l yeast extract and 10 g/l NaCl. MOPS buffered minimal medium contained 40 mM MOPS (Coolaber, Beijing), 0.1 M NaCl, 4 mM Tricine (adjusted to pH 7.4 with NaOH), 0.1 mM FeSO_4_, 0.276 mM Na_2_SO_4_, 0.5 μM CaCl_2_, 0.523 mM MgCl_2_ and also micronutrient mixtures as used in Cayley *et al.* (42). The nitrogen source used was always 10 mM NH_4_Cl. Carbon sources used included 0.2% (w/v) glucose, 0.2% (v/v) glycerol, 60 mM sodium acetate and 0.2% (w/v) mannose.

### Cell growth

Cell growth was always performed in a 37°C air bath shaker (220 rpm). The cell growth procedure consisted of three steps: seed culture, pre-culture and experimental culture. Cells from a fresh colony in the LB plate were inoculated into LB liquid medium and grew for several hours as seed culture. The seed culture was then transferred into the medium of the final experimental culture (e.g. glucose minimal medium or glycerol minimal medium) and grew overnight at 37°C as pre-culture. In the next day, the overnight pre-culture was inoculated into the same medium as pre-culture at an initial OD_600_ ≈0.015 as experimental culture. Note that for the IPTG induction of RelA^+^ expression, Mesh1 expression or SpoT E319Q expression, the IPTG inducer was only supplemented to the final experimental culture. For each condition, we took 6–8 OD_600_ points (at the range of 0.1– 0.5) to get an exponential growth curve for calculation of growth rate. The values of OD_600_ were measured by a Thermo Sci genesys30 spectrophotometer.

### Total RNA measurement

The total RNA quantification was based on the perchloric acid/potassium hydroxide method detailed in You *et al.* (13).

### Total Protein measurement

The total protein quantification was based on the Biuret method detailed in by You *et al.* (13).

### Measurement of the β-galactosidase (LacZ) activity

The LacZ assay was based on the traditional Miller's colorimetric method using *O*-nitrophenyl-β-d-galactopyranoside (ONPG) as the substrate. The detailed procedure is the same as described in You *et al.* (13).

### Measurement of translational elongation rate of *E. coli* upon RelA^+^ overexpression

The translational elongation rate measurement of *E. coli* cells was based on the classical LacZ induction assay as detailed in (9). Given that the RelA^+^ overexpression was based on the IPTG inducer, we used the TetR-P*_LtetO_* induction system to measure the translational elongation rate of *E. coli* with anhydrotetracycline (aTc) as the inducer, which was the same as described in (43). An NCM3722-derived FL94 strain with TetR-P*_LtetO_* induction system was made to measure the translational elongation rate. The *YcaC/YcaD* site of the genome of the NQ122-1 strain (*rrnBT*-P*_LtetO_*-*lacZ*) (44) was integrated into a *kmR*-P*_LtetO_*-*tetR* cassette through λ-Red recombination system, to obtain a FL71 strain (*kmR*-P*_LtetO_*-*tetR, rrnBT*-P*_LtetO_*-*lacZ*). The pLAS14-relA^+^ vector was transformed into FL71 strain to obtain FL94 strain. The FL94 cultures with different degrees of RelA^+^ overexpression were exponentially growing to OD_600_ ≈0.4, being followed by addition of 100-ng/ml aTc to induce the expression of LacZ. The LacZ induction curve was used to deduce the translational elongation rate of *E. coli* as described in (9) and (43).

### Measurement of cellular ppGpp level

The measurement of cellular guanosine tetra-phosphate (ppGpp) level was based on the UPLC-MS method as described in Yuta *et al.* (45). *Escherichia coli* cell culture was growing exponentially to OD_600_ ≈0.4. 2 ml cell sample was taken and immediately collected by centrifugation for 0.5 min at 14 000 rpm at 4°C and washed once by ice-cold water. Cells were then crushed in 3 ml pre-cooled 2 M formic acid at 30 min and subjected to solid-phase extraction (SPE) protocol as detailed in Ref. (45). The UPLC-MS experiment was performed with Thermo Scientific TSQ Fortis triple quadrupole mass spectrometer.

### Measurement of total cellular NADP(H) and NAD(H) level

The measurements of total cellular NADP(H) (the sum of NADP^+^ and NADPH) level and NAD(H) (the sum of NAD^+^ and NADH) level were based on Amplite™ Fluorimetric NADPH/NADP^+^ assay kit (cat# 15264, AAT Bioquest) and Amplite™ Fluorimetric NADH/NAD^+^ assay kit (cat# 15263, AAT Bioquest), respectively.

## RESULTS

### Selection of genetic tools for titrating the ppGpp level

**Figure 1.**
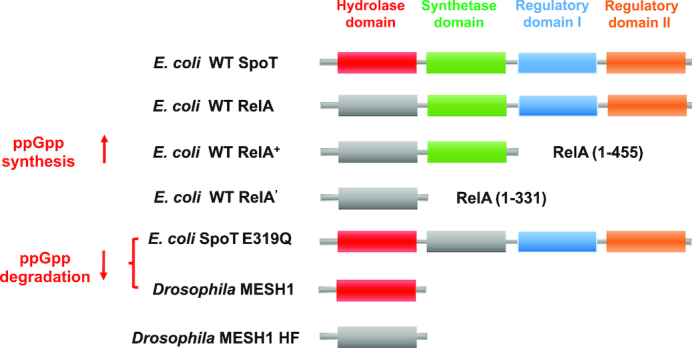
Genetic tools used for perturbing cellular ppGpp levels in this study. The wild type *E. coli* cell has two ppGpp synthetases, SpoT and RelA. SpoT possesses both ppGpp hydrolase activity and synthetase activity while RelA only has synthetase activity. Wild type SpoT protein contains four functional domains, the N-terminal hydrolase domain (red), the adjacent synthetase domain (green) and two C-terminal regulatory domains (blue and orange). RelA shares a similar topology structure with SpoT except that its hydrolase domain is inactive. The *E. coli* RelA^+^ variant contains the N-terminal 455 residues of wild type RelA protein, exhibiting constitutive ppGpp-synthesizing activity. The *E. coli* RelA' variant contains the N-terminal 331 residues of wild type RelA protein, exhibiting no ppGpp-synthesizing activity. The *E. coli* SpoT E319Q mutant has its ppGpp synthetase activity abolished due to an E319Q mutation in the synthetase domain. The Mesh1 protein is a metazoan single-function ppGpp hydrolase from *Drosophila melanogaster*. It shares a similar structure topology with the hydrolase domain of SpoT. The Mesh1 H62F point mutant has its ppGpp-hydrolyzing activity completely abolished.

To systematically investigate the role of ppGpp in regulating cell growth, it is important to establish a systematic approach for titrating (including both increasing and reducing) the ppGpp level. We first reviewed several proteins involved in ppGpp metabolism (Figure 1). In *E. coli* cells, the ppGpp level is maintained by the balance between its synthesis via RelA/SpoT and its degradation via SpoT (46). SpoT, exhibiting both ppGpp synthetase and hydrolase activities, consists of four domains including hydrolase domain, synthetase domain and two C-terminal regulatory domains (46). The wild-type RelA protein, although sharing similar structure topology with SpoT, only has synthetase activity (46). Previous studies have shown that the RelA^+^ protein, a RelA protein variant containing the N-terminal 455 amino acid residues of wild type RelA, retains constitutive ppGpp synthetase activity (30,31). Therefore, we selected RelA^+^ protein for systematical elevation of ppGpp levels. Unlike the case of ppGpp elevation, it is not straightforward to reduce ppGpp levels using SpoT since it is a ppGpp synthesis/hydrolysis bi-functional protein. To address this issue, we focused on some single-function ppGpp hydrolases. The short-length Mesh1 protein (Supp Text 1) is a single-function ppGpp hydrolase that has been recently identified in eukaryotes such as *Drosophila* and *Homo sapiens* (40,46). It was recently found that the Mesh1 protein could efficiently hydrolyze ppGpp *in vivo* (40). Therefore, we selected the Mesh1 protein to systematically lower the ppGpp levels. In addition, a recent study has shown that the SpoT E319Q variant abolishes the ppGpp synthetase activity while retains the ppGpp hydrolase activity (47), thus providing another potential genetic approach for reducing ppGpp levels.

### Growth and ribosome content at increased ppGpp levels

**Figure 2.**
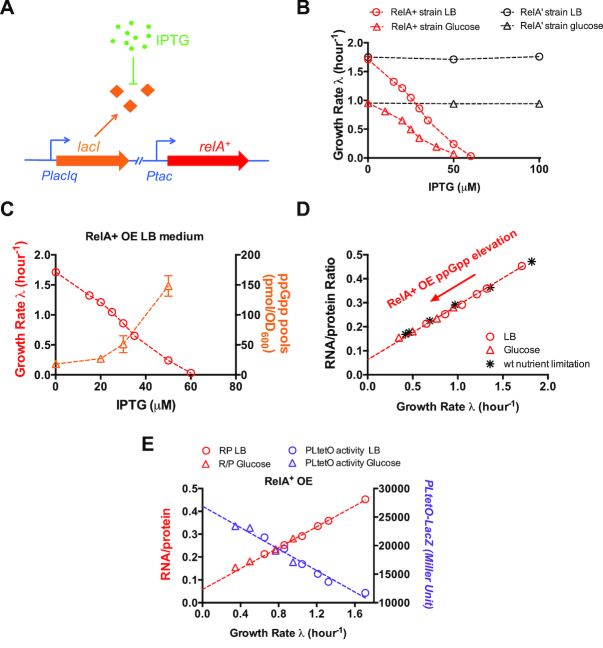
The effect of increased ppGpp levels on growth rate, ribosome content and constitutive gene expression in *E. coli*. (**A**) The *relA^+^* gene is driven by *Ptac* promoter under the control of *PlacIq-lacI* cassette. (**B**) Effect of RelA^+^ overexpression (OE) on the growth rate of *E. coli* in LB medium and glucose minimal medium. (**C**) The cellular guanosine tetraphosphate (ppGpp) level of *E. coli* upon RelA^+^ OE. The standard deviation of ppGpp measurement by UPLC-MS is within 25%. (**D**) Ribosome content (RNA/protein ratio) plotted against growth rate under different degrees of RelA^+^ protein overexpression. Data of nutrient limitation denotes the data of wild type cells growing in six different nutrient conditions including LB medium, glucose casamino acid medium, glucose minimal medium, glycerol minimal medium, acetate minimal medium and mannose minimal medium. (**E**) The constitutive *PLtetO-lacZ* activity plotted against growth rate under different degrees of RelA^+^ protein overexpression. Data are average of triplicates with standard deviations being within 10% (approximately the size of symbol).

We first systematically investigated the effect of increased ppGpp levels on cell growth. Previous studies have shown that overexpression of the constitutively active RelA^+^ protein by *Ptac* system causes dramatic elevation in the cellular ppGpp level (30,31). We used the IPTG inducible *Ptac* promoter to drive the expression of *relA^+^* in wild type *E. coli* K-12 NCM3722 strain (Figure 2A). The growth rate of *E. coli* (λ) decreases strongly with increased IPTG concentrations in both LB medium and glucose minimal medium (red symbols in Figure 2B, Supplementary Figure S1A). Cell growth is completely arrested at IPTG >60 μM. We further found that knockout of *lac* operon in wild type cells did not affect the IPTG-dependent pattern of growth rate upon RelA^+^ overexpression (blue symbols in Supplementary Figure S2A), indicating that the presence of LacY and LacA in wild type cells did not affect the intracellular IPTG level. In contrast, when we overexpressed an inactive RelA variant, RelA’ consisting of the N-terminal 331 residues of the native RelA protein (30,31), no slow-down of growth was observed even at 100 μM IPTG (black symbols in Figure 2B). This result demonstrates that it is the increased ppGpp level that causes slow-down of cell growth. Direct measurement with UPLC-MS showed that the cellular ppGpp level indeed increased dramatically upon RelA^+^ overexpression (orange triangles in Figure 2C). To understand the origin of growth suppression under increased ppGpp levels, we characterized the cellular ribosome content (which could be accurately and quantitatively reflected by RNA/protein ratio, *R/P*, being proportional to cellular ribosomal protein abundance in various conditions) (3,9,11,48), which is the major demand of cell growth (12). The ribosome content exhibits an intriguingly linear correlation with growth rate (Figure 2D, red symbols), which is consistent with previous findings that ppGpp overproduction inhibits rRNA synthesis (31). This linear correlation under ppGpp overproduction shares almost the same pattern as that of wild type cells growing in different nutrient conditions (Figure 2D, black asterisks). As it is well known that ribosome abundance is central for cell growth under nutrient limitation (2,3,12), our result strongly supports that increased ppGpp levels suppresses cell growth through inhibiting the ribosome synthesis, as has also been found in the stringent response (21). On the other hand, our results reinforce the notion that the growth-rate dependent control of ribosome synthesis under nutrient limitation can be attributed to the change in ppGpp levels (3,9,11,49). To gain an additional insight into the effect of ppGpp overproduction on ribosome activity, we also measured the translational elongation rate (ER) of ribosome upon RelA^+^ overexpression. ER keeps constant at fast growth conditions (*λ* > 1/h) but drops slightly at slower growth (Supplementary Figure S3B), being again similar with the pattern found previously under nutrient limitation (9). The data of RNA/protein ratio and ER, together, allow us to deduce the active ribosome fraction of *E. coli* upon ppGpp overproduction. As shown in Supplementary Figure S3C, the active ribosome fraction keeps at ∼85%, suggesting that ppGpp overproduction does not significantly inhibit ribosome maturation in our growth conditions.

Since ribosome content decreases strongly upon ppGpp overproduction, we expected that some other proteins should simultaneously increase from the perspective of proteome resource allocation (2). The unregulated (constitutively expressed) proteins belong to a set of proteins being subject to different modes of control from ribosomes (3,50). For example, the expression of a constitutive promoter increases under nutrient limitation, being opposite to the pattern of ribosome content and mimicking the behavior of metabolic proteins (3,13). We thus measured the expression of the constitutive *PLtetO* promoter upon ppGpp overproduction. As expected, the expression of the *PLtetO*-*lacZ* indeed increases linearly with decreasing growth rate (Figure 2E, purple symbols), exhibiting an opposite pattern to ribosome content. The pattern of *PLtetO-lacZ* activity upon ppGpp overproduction is also similar to that of wild type cells growing under nutrient limitation (13).

### Growth and ribosome content at decreased ppGpp levels

**Figure 3.**
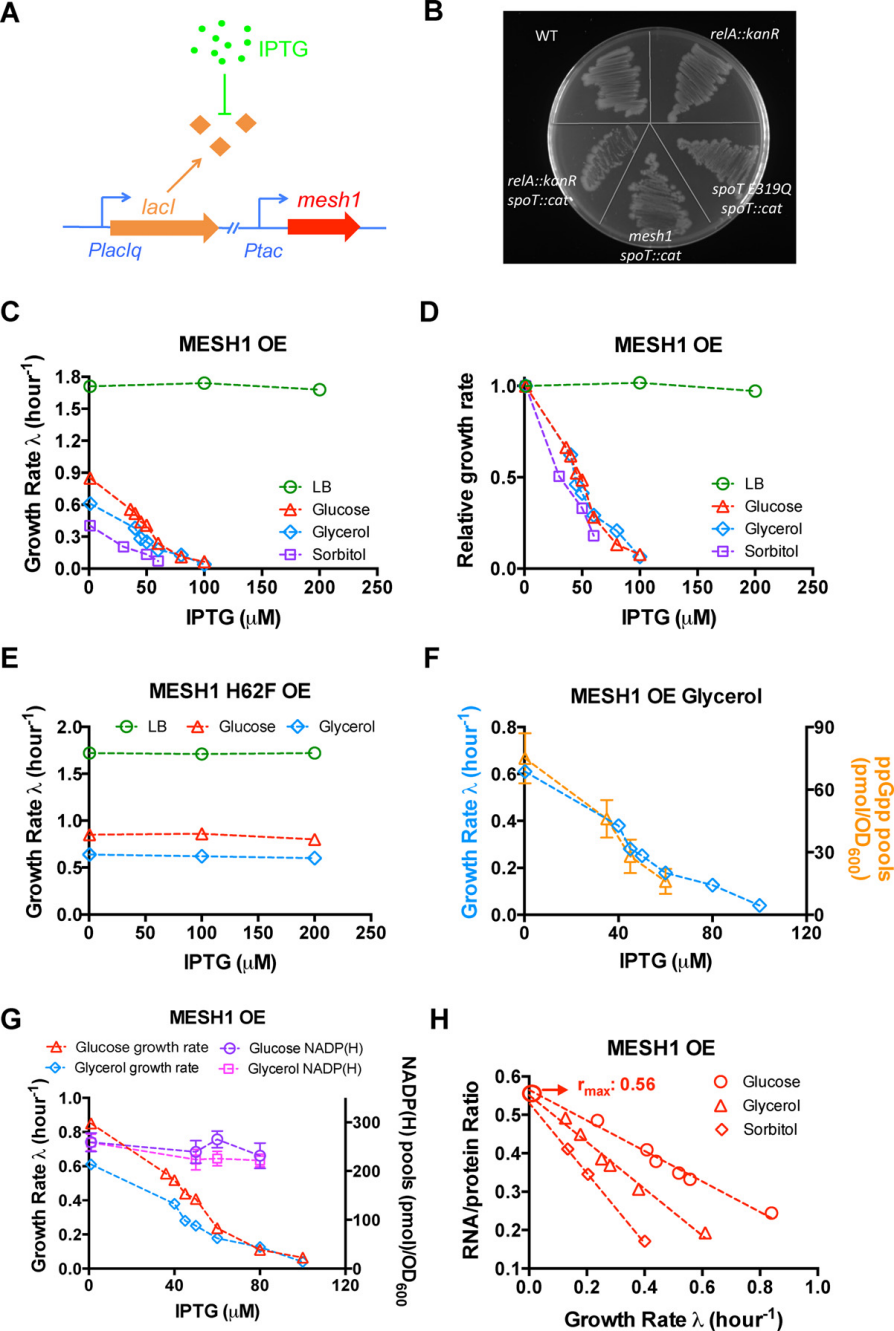
Lowering ppGpp levels in *E. coli* by overexpression (OE) of Mesh1 protein from *Drosophila melanogaster*. (**A**) The *mesh1* gene is driven by *Ptac* promoter under the control of *PlacIq-lacI* cassette. (**B**) Expression of Mesh1 or SpoT E319Q in *E. coli* can suppress the lethal effect of SpoT knockout. The LB plate was supplemented with 30 μM IPTG to induce the expression of Mesh1 or SpoT E319Q. (**C**) Effect of Mesh1 protein overexpression on the growth rate of wild type *E. coli* cells in LB medium, glucose medium, glycerol medium and sorbitol medium. (**D**) The relative change of growth rate of wild type *E. coli* cells upon Mesh1 protein overexpression in each growth condition. The growth rate data of no-IPTG in each condition in panel C is set as ‘1’. (**E**) Effect of overexpression of Mesh1 H62F mutant protein on the growth rate of wild type *E. coli* cells in LB medium and three minimal media. (**F**) The cellular guanosine tetraphosphate (ppGpp) level of *E. coli* growing in glycerol minimal medium upon Mesh1 OE. The standard deviation of ppGpp measurement by UPLC-MS is within 25%. (**G**) The cellular NADP(H) pool of *E. coli* cells under Mesh1 overexpression. Cells were growing in either glucose minimal medium or glycerol minimal medium. The growth rate data in glucose and glycerol medium is exactly the same as shown in panel C. (**H**) Ribosome content (RNA/protein ratio) plotted against growth rate under Mesh1 protein overexpression. The *r*_max_ denotes the offset of Y-axis when growth rate becomes zero. Data are average of triplicates with standard deviations being within 10%.

We next investigated the effect of decreased ppGpp levels on cell growth and gene expression. A recent study has identified the Mesh1 protein, which is a metazoan SpoT ortholog found in both *Drosophila* and *Homo sapiens* (40). Thin-layer chromatography (TLC) study has shown that expression of Mesh1 protein by *Ptac* promoter could effectively reduce ppGpp levels *in vivo* (40). Therefore, we used the IPTG inducible *Ptac* promoter to control the expression of *mesh1* gene from *D. melanogaster* (Figure 3A) (Supp Text 1). The expression of Mesh1 in *E. coli* allowed the knockout of *spoT* gene of *E. coli* (Figure 3B), confirming that Mesh1 could effectively hydrolyze ppGpp *in vivo* since *spoT* knockout in wild type cells is lethal due to the uncontrollably ppGpp accumulation via RelA protein (51,52). We then transformed the *mesh1* inducible vector into the wild type NCM3722 strain. The growth of *E. coli* is severely inhibited with increasing IPTG levels in three amino-acid free media with glucose, glycerol and sorbitol as the sole carbon source, respectively (red triangle, blue diamond and purple square in Figure 3C and D, Supplementary Figure S1B). The IPTG-dependent pattern of growth rate of cells in glucose and glycerol medium upon Mesh1 overexpression is not affected by the deletion of *lac* operon of wild type cells (purple asterisk and green circles in Supplementary Figure S2B), again indicating that the presence of LacY and LacA in wild type cells does not significantly affect the intracellular IPTG levels. In contrast, *E. coli* cells overexpressing the Mesh1 H62F protein, a Mesh1 mutant abolishing the ppGpp-hydrolyzing activity (40), exhibit no growth defects in all the growth conditions (Figure 3E). Being different from the case of amino acid-free minimal medium, the growth rates of Mesh1-expressing *E. coli* cells in LB medium and rich defined medium (RDM) change only slightly even at 200 and 500 μM IPTG (green circles of Figure 3C, 3D; Supplementary Figure S4A; purple circles of Supplementary Figure S5). This observation is reminiscent of the phenotype of Δ*relA*Δ*spoT* amino acid-auxotrophic ppGpp-null strain, which grows in LB medium with a slightly reduced growth rate compared with the wild type strain (Supplementary Figure S4B) but cannot grow in minimal medium (32,33). Direct measurement by UPLC-MS clearly showed that the cellular ppGpp level of *E. coli* decreased dramatically with decreasing growth rate upon Mesh1 overexpression (orange triangles of Figure 3F). In addition, *E. coli* cells overexpressing Mesh1 in LB medium also exhibit filament morphology during stationary phase, as has been previously observed for Δ*relA*Δ*spoT* ppGpp-null strain (Supplementary Figure S6A-B) (53). Overall, those various pieces of evidences demonstrate that Mesh1 overexpression strongly suppresses cell growth in amino acid-free minimal medium through dramatically lowering the cellular ppGpp level. It was recently suggested that Mesh1 can act as a NADPH phosphatase in mammalian cells (54). However, no significant change in the levels of either NADP(H) (Figure 3G) or NAD(H) pools (Supplementary Figure S7) of *E. coli* was observed upon Mesh1 overexpression in our growth conditions, indicating that the expression levels of Mesh1 in our study does not disrupt the cellular homeostasis of NADP(H)/NAD(H) pools of *E. coli* cells.

It is intriguing that decreased ppGpp levels also causes similar systematic slow-down of cell growth as found in the case of increased ppGpp levels. To investigate the origin of growth slow-down, we again measured the cellular ribosome content. A recent work has found that the ppGpp-null strain exhibits loss of growth rate control of ribosome synthesis in amino-acid supplemented medium (32). However, two earlier studies found contradictory results (35,36), showing that ppGpp-null strain still exhibits similar growth-rate dependent ribosome content as wild-type cells, making the situation being complicated (27). Here, the Mesh1 system allows us to systematically investigate the effect of decreased ppGpp levels on ribosome content in minimal medium in which the ribosome content of wild type cells is low (9). The ribosome content increases strongly under decreased ppGpp levels, being again linearly but negatively correlated with growth rate (Figure 3H). The pattern is opposite to that observed under increased ppGpp level (Figure 2D), further clarifying the crucial role of ppGpp in regulating ribosome synthesis during steady-state growth. We further deduced the proteome abundance of ribosomal affiliated proteins in order to estimate how much proteome resource of *E. coli* cell invests on the ribosome synthesis under decreased ppGpp levels. Quantitatively, the maximal *R/P* value equals to 0.56 at zero growth (the offset in y-axis of Figure 3H), corresponding to a maximal proteome fraction of ribosome affiliated proteins, *ϕ*_*R*_ = 43% (based on ${\phi _R} = \ R/P \cdot \rho$, *ρ* = 0.76 as deduced in (3)) under extreme shortage of ppGpp pools.

To further reinforce the finding using Mesh1 system, we also applied the *E. coli*-origin SpoT E319Q variant to perturb ppGpp levels using the inducible *Ptac* system (Supplementary Figure S8B). SpoT E319Q is a variant of SpoT abolishing its ppGpp synthetase activity and is thus also a single-function ppGpp hydrolase (47). Supporting the above statement, the expression of SpoT E319Q also allows the knockout of *spoT* gene in wild type *E. coli* (Figure 3B). We further found that SpoT E319Q overexpression led to a moderate growth slow-down of wild type cells in glucose medium (red asterisk in Supplementary Figure S8C and D), indicating its weaker capacity of hydrolyzing ppGpp than Mesh1 protein. However, SpoT E319Q overexpression causes dramatic growth slow-down in glucose medium when the native *relA* ppGpp synthetase gene is deleted (red circles in Supplementary Figure S8C and S8D). Being similar with the case of Mesh1, the strong growth suppression only occurs in glucose medium, not in LB rich medium (Supplementary Figure S8C and S8D, black symbols). Since the wild type cells exhibit no differences in growth rate and ribosome content compared with Δ*relA* mutant under nutrient limitation (Supplementary Figure S8A), we performed SpoT E319Q overexpression in both the *ΔrelA* background and the wild type background. We found a similar strong increase in ribosome content upon decreased ppGpp levels via SpoT E319Q overexpression (Supplementary Figure S9A), being consistent with the result of Mesh1 overexpression.

### Expression of constitutive and metabolic promoters at decreased ppGpp levels

**Figure 4.**
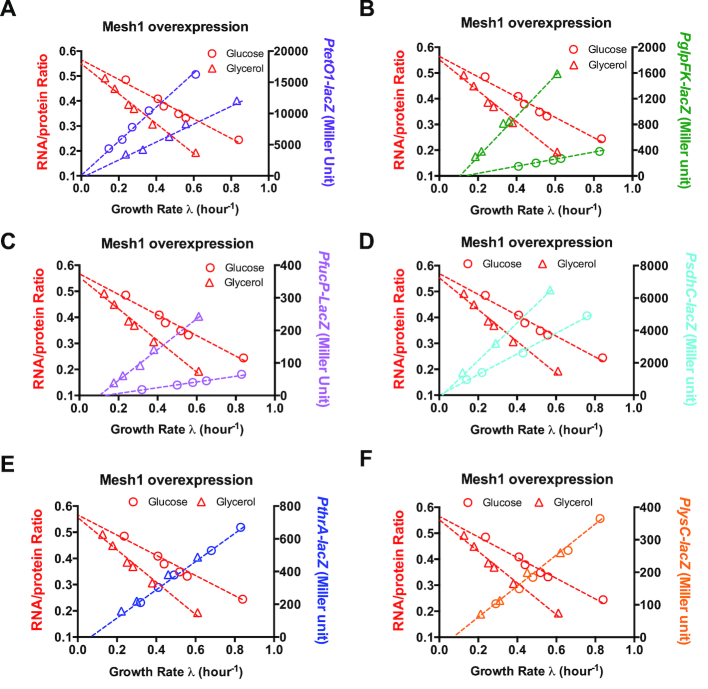
The effect of decreased ppGpp levels on gene expression of *E. coli*. Experiments were preformed for cells growing in either glucose minimal medium (circle) or glycerol minimal medium (triangle). The activity of six promoters plotted against growth rate under different degrees of Mesh1 protein overexpression. (**A**) *PtetO* promoter; (**B**) *PglpFK* promoter; (**C**) *PfucP* promoter; (**D**) *PsdhC* promoter; (**E**) *PthrA* promoter; (**F**) *PlysC* promoter. Data are average of triplicates with standard deviations being within 10%.

Our result demonstrates that ppGpp overproduction reduces growth rate through inhibiting the ribosome synthesis. However, cell growth is also strongly suppressed in minimal medium at decreased ppGpp levels in which ribosome content increases. Then what could account for the growth slow-down at decreased ppGpp levels? We consider this issue from the perspective of resource allocation. When growing in minimal medium, cells must allocate a certain proteome fraction into metabolic proteins (including catabolism and anabolism), which are indispensable for nutrient uptake and metabolism. However, the increased ribosome content upon decreased ppGpp levels may compress the proteome fraction of metabolic proteins. To test this hypothesis, we first measured the expression of the constitutively expressed *PLtetO* promoter. The *PLtetO-lacZ* activity indeed decreases strongly at decreased ppGpp levels, exhibiting a positive linear correlation with growth rate (purple symbols of Figure 4A). We further measured the expression of five metabolic promoters, *PglpFK-lacZ, PfucP-lacZ, PsdhC-lacZ, PlysC-lacZ* and *PthrA*-*lacZ*. The *PglpFK, PfucP* and *PsdhC* are three promoters of the catabolic operons, *glpFK* (glycerol metabolism), *fucPIK* (fuctose metabolism) and *sdhCDAB*-*sucABCD* (succinate metabolism), respectively, which all belong to the CRP regulon (13,55). The *PthrA* and *PlysC* are two anabolic promoters for threonine biosynthesis and lysine biosynthesis, respectively (56,57). For all the five metabolic promoters, only *PthrA* has been known to be positively regulated by ppGpp (25). Strikingly, the activities of all the five metabolic promoters decrease strongly upon ppGpp decreasing, being again linearly correlated with the growth rate (Figure 4B–F). Similar results have been observed when we used SpoT E319Q protein to lower the ppGpp level (Supplementary Figure S9B-D). These results demonstrate that decreased ppGpp levels up-regulate the ribosome synthesis at the expenses of metabolic proteins. Therefore, compared to the normal growth condition, the altered ppGpp levels result in a non-optimal resource allocation with increased ppGpp levels limiting ribosome synthesis and decreased ppGpp levels limiting the expression of metabolic proteins.

## DISCUSSION

**Figure 5.**
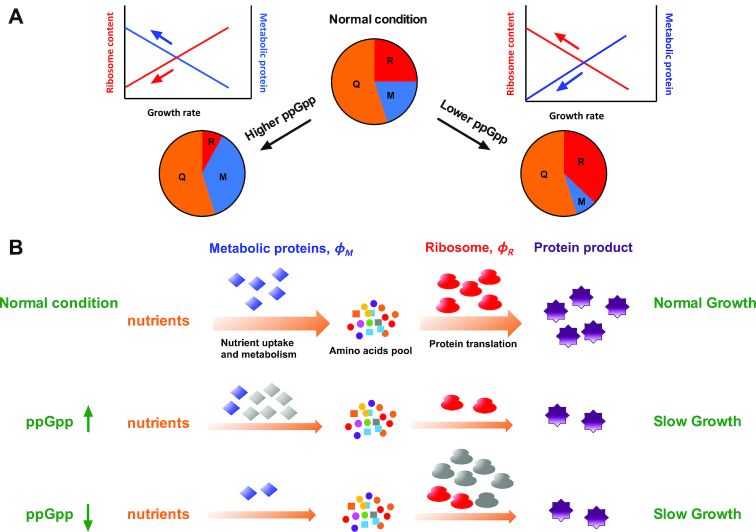
Growth control by ppGpp via governing global resource allocation in *E. coli*. (**A**) ppGpp mediates the proteome resource allocation among metabolic proteins and ribosome-affiliated proteins. In a recently proposed coarse-grained model, the *E. coli* proteome is partitioned into three sectors, R-sector including ribosome proteins and related affiliated protein (e.g. EF-Tu, EF-G), M-sector including metabolic proteins (for nutrient processing) and the constant growth-rate independent Q-sector. At higher ppGpp level, R-sector becomes smaller while M-sector becomes larger. In contrast, lower ppGpp level leads to higher R-sector but smaller M-sector. (**B**) Coarse-grained schematics from nutrients metabolism to protein synthesis. The external nutrients need to be assimilated and metabolized by metabolic proteins (belonging to M-sector, *ϕ_M_*) to generate amino acids, which is further polymerized into protein product by the ribosomes (belongs to R-sector, *ϕ_R_*). The flux from metabolism to protein synthesis is balanced in a steady-state growth. Compared with normal condition, higher ppGpp limits the protein synthesis flux (due to lower *ϕ_R_*), while lower ppGpp level limits the metabolic flux (due to lower *ϕ_M_*), both causing slower growth rate compared with normal condition. The gray color means that metabolic proteins (at higher ppGpp case) and ribosomes proteins (at lower ppGpp case) become unnecessarily high (like ‘useless’ protein overexpression). The flux (orange arrow) for biomass growth becomes smaller in altered ppGpp levels due to either smaller *ϕ_R_* or *ϕ_M_*, leading to slower overall protein synthesis rate (purple gears) and cell growth.

The importance of maintaining an optimal ppGpp level for cell growth is a fundamental question. However, studies on the connection of ppGpp to steady-state cell growth have been severely hindered by the technical limitations of ppGpp-null strain. In this study, the combination between RelA^+^ and Mesh1 system provides a powerful genetic approach to systematically investigate the effect of ppGpp perturbation on bacterial growth physiology. Specially, the introduction of Mesh1 systems allows us to systematically investigate the effect of lowering ppGpp levels on cell growth and gene expression in amino-acid free minimal medium, which could not be done traditionally with ppGpp-null strain. We found that increased and decreased ppGpp levels both suppress cell growth through limiting either the ribosome synthesis or the expression of metabolic proteins. Below we highlight the role of ppGpp in steady-state cell growth in light of a recently developed phenomenological resource-allocation model by Hwa and colleagues (2,3,10). In this model, the *E. coli* proteome is partitioned into three coarse-grained sectors, a fraction including ribosome-affiliated proteins ${\phi _R}$, a fraction of metabolic proteins (including constitutively expressed proteins), ${\phi _M}$ and a growth-rate independent fraction, $\ {\phi _Q}$ (Figure 5A). The constancy of ${\phi _Q}$ could be achieved through an auto-negative regulatory feedback mechanism (3,10). ${\phi _R}$ and ${\phi _M}$ both exhibit linear correlations with biomass growth rate$\ \lambda$ (1–3,12,13), as described by:

(1) \begin{equation*}\lambda = \gamma \cdot {\phi _R}\end{equation*}

(2) \begin{equation*}\lambda = \nu \cdot {\phi _M}\end{equation*}

where $\gamma$ refers to the translational efficiency (describing the amount of ribosome proteins needed to attain a certain rate of total protein synthesis), and $\nu$ being the metabolic efficiency (describing the amount of metabolic proteins needed to attain a certain metabolic flux). ${\phi _R}$ and ${\phi _M}$ is further related by the following constraint (Equation 3),

(3) \begin{equation*}{\phi _R} + \ {\phi _M} = {\phi _{\max }},\end{equation*}

where ${\phi _{\max }} = 1 - {\phi _{Q\ }}$, being the total proteome fraction linked to biomass growth (including ribosome proteins and metabolic proteins, Equation 3) and equaling to ∼40% of the *E. coli* proteome based on measurement by either quantitative mass spectrometry or protein overexpression (1,3,6,13).

For steady-state growth, the flux (orange arrow of Figure 5B) from nutrient uptake and metabolism (done by ${\phi _M}$) to protein translation (done by ${\phi _R}$) is balanced to support biomass growth (illustrated by Figure 5B, normal condition). However, the optimal resource allocation is broken by altered ppGpp levels. At higher ppGpp levels, ribosome fraction, ${\phi _R}$ drops substantially (Figure 2D and Figure 5A), further limiting the flux for biomass growth $\lambda$ (Equation 1, illustrated by Figure 5B, ppGpp↑). This picture is similar with the case of nutrient limitation (Figure 2D, black asterisks), where ribosome content limits the cell growth in poor nutrients (2,3). At lower ppGpp levels, ${\phi _R}$ becomes unnecessarily higher due to the loss of ribosome control, compressing the fraction of metabolic proteins, ${\phi _M}$ (Figures 4 and 5A), leading to smaller flux for biomass growth $\lambda$ (Equation 2, illustrated by Figure 5B, ppGpp↓). Quantitatively, ${\phi _R} = \ 43\%$ at $\lambda \ = \ 0$ during lowering ppGpp levels (Figure 3G), being equal to ${\phi _{max}}$. In this case, ${\phi _M} \to 0$, as confirmed by the data of Figure 4. Therefore, the resource allocation model quantitatively accounts for the regulation of ppGpp on exponential growth through balancing the synthesis of ribosome and metabolic proteins.

It is well known that ppGpp overproduction during stringent response (e.g. amino acid starvation) could suppress ribosome synthesis and instead active the expression of amino acid biosynthetic promoters (18,24,25). However, our study on lowering ppGpp levels quantitatively reveals that the effect of ppGpp on gene expression is not merely limited to amino acid biosynthesis promoters and instead occurs at a much larger scale including catabolic proteins and even unregulated, constitutively expressed proteins (Figure 4A–D), which have not been known previously to be regulated by ppGpp. The inhibited expression of constitutive protein and catabolic protein under decreased ppGpp levels does not require a direct role of ppGpp in regulating specific promoters like amino acid biosynthesis promoters (25). Instead, it occurs in a broader scale due to the resource competition from the up-regulated expression of ribosome since up-regulation of ribosome synthesis requires a larger fraction of ribosomes to synthesize ribosomal proteins themselves so that a smaller fraction of ribosome is available to synthesize other proteins (2,10). Quantitatively, for the six promoters, only *PthrA* (Figure 4E) is known to be directly activated by ppGpp during stringent response (25). There are no obvious differences in the behavior of *PthrA* from other five promoters (including unregulated *Ptet*) at decreased ppGpp levels. Given that the activating effect of *PthrA* by ppGpp is mainly found during stringent response (e.g. amino acid starvation conditions) while we are studying steady-state cell growth here, this observation suggests that resource competition plays a dominant role in the effect of decreased ppGpp levels on gene expression. In the extreme condition of ppGpp shortage where growth stops (Figure 3H), the proteome abundance of ribosome affiliated proteins ( ${\phi _R} = \ 43\%$) could occupy all the space of the *E. coli* proteome that linked to biomass growth (${\phi _{max}}$), leaving no metabolic proteins (including catabolic protein and amino acid biosynthesis proteins) to support cell growth. This result quantitatively accounts for the amino acid auxotrophy and inability of ppGpp-null strain to grow in minimal medium. Overall, suppression of cell growth by altered ppGpp levels results from a non-optimal resource allocation between ribosome fraction and metabolic protein fraction (not limited to amino acid biosynthesis proteins) in *E. coli*. A recent phenomenological study has revealed the profound role of cAMP in mediating *E. coli* resource allocation between catabolic proteins and anabolic proteins (13). Here, we reveal a global role of ppGpp in regulating bacterial growth through governing resource allocation between ribosomes and metabolic proteins. Those two studies together suggest that *E. coli* employs simple and predictable global signaling pathways to manipulate its global expression pattern.

Researchers in the field of ppGpp physiology rely strongly on the ppGpp-null (Δ*relA*Δ*spoT*) strain (21,33,58–60). However, severe technical drawbacks are associated with the use of such strain. First, the ppGpp-null strain can only grow in rich medium such as LB broth. The ppGpp level is already very low in wild type cells growing in rich medium but increases strongly in minimal medium (11,37). Therefore, the physiological functions of ppGpp revealed from ppGpp-null strain growing in rich medium might be limited or might not be applicable to cells growing in poor conditions. Moreover, the ppGpp-null strain suffers viability loss in stationary phase and can easily obtain suppressor mutations (21,34), thus requiring tedious efforts to monitor the status of its growth. In addition, because of the genetic instability and poor survivability of ppGpp-null strain, researchers need to frequently perform P1 transduction to remake fresh strains. The technical limitation of ppGpp-null strain has even led to some significant controversies regarding the regulation of ribosome synthesis by ppGpp under steady-state growth (32,35,36). Our study using the Mesh1 system clearly shows that lowering the ppGpp levels dramatically up-regulates the ribosome content of *E. coli* in minimal medium (Figure 3G), together with the data of RelA^+^ system (Figure 2D), clarifying the central role of ppGpp in controlling ribosome synthesis during steady-state growth. Given that the Mesh1 protein is a metazoan-origin protein, although we did not observe significant change in the NADP(H) level, one may still argue that Mesh1 might have some other unknown targets in *E. coli*. However, the consistency between data obtained with Mesh1 protein and *E. coli*-origin SpoT E319Q protein has greatly eased such concern. In general, the use of Mesh1 ppGpp hydrolase from *Drosophila melanogaster* successfully overcomes those drawbacks of ppGpp-null strain, highlighting itself as a powerful genetic tool for interrogating bacterial ppGpp physiology in future studies.

## Supplementary Material

Supplementary Data

## References

[1] Hui S., Silverman J.M., Chen S.S., Erickson D.W., Basan M., Wang J., Hwa T., Williamson J.R. Quantitative proteomic analysis reveals a simple strategy of global resource allocation in bacteria. Mol. Syst. Biol. 2015; 11:784. 25678603 10.15252/msb.20145697 PMC4358657

[2] Scott M., Klumpp S., Mateescu E.M., Hwa T. Emergence of robust growth laws from optimal regulation of ribosome synthesis. Mol. Syst. Biol. 2014; 10:747. 25149558 10.15252/msb.20145379 PMC4299513

[3] Scott M., Gunderson C.W., Mateescu E.M., Zhang Z., Hwa T. Interdependence of cell growth and gene expression: origins and consequences. Science. 2010; 330:1099–1102. 21097934 10.1126/science.1192588

[4] Gerosa L., Kochanowski K., Heinemann M., Sauer U. Dissecting specific and global transcriptional regulation of bacterial gene expression. Mol. Syst. Biol. 2013; 9:658. 23591774 10.1038/msb.2013.14 PMC3658269

[5] Li G.W., Burkhardt D., Gross C., Weissman J.S. Quantifying absolute protein synthesis rates reveals principles underlying allocation of cellular resources. Cell. 2014; 157:624–635. 24766808 10.1016/j.cell.2014.02.033 PMC4006352

[6] Basan M., Hui S., Okano H., Zhang Z., Shen Y., Williamson J.R., Hwa T. Overflow metabolism in Escherichia coli results from efficient proteome allocation. Nature. 2015; 528:99–104. 26632588 10.1038/nature15765 PMC4843128

[7] Metzl-Raz E., Kafri M., Yaakov G., Soifer I., Gurvich Y., Barkai N. Principles of cellular resource allocation revealed by condition-dependent proteome profiling. Elife. 2017; 6:e28034. 28857745 10.7554/eLife.28034 PMC5578734

[8] Keren L., Hausser J., Lotan-Pompan M., Vainberg Slutskin I., Alisar H., Kaminski S., Weinberger A., Alon U., Milo R., Segal E. Massively parallel interrogation of the effects of gene expression levels on fitness. Cell. 2016; 166:1282–1294. 27545349 10.1016/j.cell.2016.07.024

[9] Dai X., Zhu M., Warren M., Balakrishnan R., Patsalo V., Okano H., Williamson J.R., Fredrick K., Wang Y.-P., Hwa T. Reduction of translating ribosomes enables Escherichia coli to maintain elongation rates during slow growth. Nat. Microbiol. 2016; 2:16231. 27941827 10.1038/nmicrobiol.2016.231 PMC5346290

[10] Scott M., Hwa T. Bacterial growth laws and their applications. Curr. Opin. Biotechnol. 2011; 22:559–565. 21592775 10.1016/j.copbio.2011.04.014 PMC3152618

[11] Bremer H., Dennis P.P. Neidhardt FC Modulation of chemical composition and other parameters of the cell at different exponential growth rates. Escherichia coli and Salmonella. 1996; 2nd edn. Washington,DC Am. Soc. Microbiol 1553–1569. 10.1128/ecosal.5.2.3 26443740

[12] Klumpp S., Scott M., Pedersen S., Hwa T. Molecular crowding limits translation and cell growth. Proc. Natl. Acad. Sci. U.S.A. 2013; 110:16754–16759. 24082144 10.1073/pnas.1310377110 PMC3801028

[13] You C., Okano H., Hui S., Zhang Z., Kim M., Gunderson C.W., Wang Y.P., Lenz P., Yan D., Hwa T. Coordination of bacterial proteome with metabolism by cyclic AMP signalling. Nature. 2013; 500:301–306. 23925119 10.1038/nature12446 PMC4038431

[14] Mostertz J., Scharf C., Hecker M., Homuth G. Transcriptome and proteome analysis of Bacillus subtilis gene expression in response to superoxide and peroxide stress. Microbiology. 2004; 150:497–512. 14766928 10.1099/mic.0.26665-0

[15] Schmidt A., Kochanowski K., Vedelaar S., Ahrne E., Volkmer B., Callipo L., Knoops K., Bauer M., Aebersold R., Heinemann M. The quantitative and condition-dependent Escherichia coli proteome. Nat. Biotechnol. 2016; 34:104–110. 26641532 10.1038/nbt.3418 PMC4888949

[16] Weber A., Kogl S.A., Jung K. Time-dependent proteome alterations under osmotic stress during aerobic and anaerobic growth in Escherichia coli. J. Bacteriol. 2006; 188:7165–7175. 17015655 10.1128/JB.00508-06 PMC1636219

[17] Tao H., Bausch C., Richmond C., Blattner F.R., Conway T. Functional genomics: expression analysis of Escherichia coli growing on minimal and rich media. J. Bacteriol. 1999; 181:6425–6440. 10515934 10.1128/jb.181.20.6425-6440.1999 PMC103779

[18] Hauryliuk V., Atkinson G.C., Murakami K.S., Tenson T., Gerdes K. Recent functional insights into the role of (p)ppGpp in bacterial physiology. Nat. Rev. Microbiol. 2015; 13:298–309. 25853779 10.1038/nrmicro3448 PMC4659695

[19] Dalebroux Z.D., Swanson M.S. ppGpp: magic beyond RNA polymerase. Nat. Rev. Microbiol. 2012; 10:203–212. 22337166 10.1038/nrmicro2720 PMC13198741

[20] Magnusson L.U., Farewell A., Nystrom T. ppGpp: a global regulator in Escherichia coli. Trends Microbiol. 2005; 13:236–242. 15866041 10.1016/j.tim.2005.03.008

[21] Potrykus K., Cashel M. (p)ppGpp: still magical. Annu. Rev. Microbiol. 2008; 62:35–51. 18454629 10.1146/annurev.micro.62.081307.162903

[22] Srivatsan A., Wang J.D. Control of bacterial transcription, translation and replication by (p)ppGpp. Curr. Opin. Microbiol. 2008; 11:100–105. 18359660 10.1016/j.mib.2008.02.001

[23] Wang J.D., Sanders G.M., Grossman A.D. Nutritional control of elongation of DNA replication by (p)ppGpp. Cell. 2007; 128:865–875. 17350574 10.1016/j.cell.2006.12.043 PMC1850998

[24] Durfee T., Hansen A.M., Zhi H., Blattner F.R., Jin D.J. Transcription profiling of the stringent response in Escherichia coli. J. Bacteriol. 2008; 190:1084–1096. 18039766 10.1128/JB.01092-07 PMC2223561

[25] Paul B.J., Berkmen M.B., Gourse R.L. DksA potentiates direct activation of amino acid promoters by ppGpp. Proc. Natl. Acad. Sci. U.S.A. 2005; 102:7823–7828. 15899978 10.1073/pnas.0501170102 PMC1142371

[26] Traxler M.F., Summers S.M., Nguyen H.T., Zacharia V.M., Hightower G.A., Smith J.T., Conway T. The global, ppGpp-mediated stringent response to amino acid starvation in Escherichia coli. Mol. Microbiol. 2008; 68:1128–1148. 18430135 10.1111/j.1365-2958.2008.06229.x PMC3719176

[27] Gourse R.L., Chen A.Y., Gopalkrishnan S., Sanchez-Vazquez P., Myers A., Ross W. Transcriptional Responses to ppGpp and DksA. Annu. Rev. Microbiol. 2018; 72:163–184. 30200857 10.1146/annurev-micro-090817-062444 PMC6586590

[28] Ross W., Sanchez-Vazquez P., Chen A.Y., Lee J.H., Burgos H.L., Gourse R.L. ppGpp binding to a site at the RNAP-DksA interface accounts for its dramatic effects on transcription initiation during the stringent response. Mol. Cell. 2016; 62:811–823. 27237053 10.1016/j.molcel.2016.04.029 PMC4912440

[29] Monod J. The growth of bacterial cultures. Annu. Rev. Microbiol. 1949; 3:371–394.

[30] Svitil A.L., Cashel M., Zyskind J.W. Guanosine tetraphosphate inhibits protein synthesis in vivo. A possible protective mechanism for starvation stress in Escherichia coli. J. Biol. Chem. 1993; 268:2307–2311. 8428905

[31] Schreiber G., Metzger S., Aizenman E., Roza S., Cashel M., Glaser G. Overexpression of the relA gene in Escherichia coli. J. Biol. Chem. 1991; 266:3760–3767. 1899866

[32] Potrykus K., Murphy H., Philippe N., Cashel M. ppGpp is the major source of growth rate control in E. coli. Environ. Microbiol. 2011; 13:563–575. 20946586 10.1111/j.1462-2920.2010.02357.x PMC4556285

[33] Xiao H., Kalman M., Ikehara K., Zemel S., Glaser G., Cashel M. Residual guanosine 3′,5′-bispyrophosphate synthetic activity of relA null mutants can be eliminated by spoT null mutations. J. Biol. Chem. 1991; 266:5980–5990. 2005134

[34] Murphy H., Cashel M. Isolation of RNA polymerase suppressors of a (p)ppGpp deficiency. Methods Enzymol. 2003; 371:596–601. 14712731 10.1016/S0076-6879(03)71044-1

[35] Gaal T., Gourse R.L. Guanosine 3′-diphosphate 5′-diphosphate is not required for growth rate-dependent control of rRNA synthesis in Escherichia coli. Proc. Natl. Acad. Sci. U.S.A. 1990; 87:5533–5537. 2196571 10.1073/pnas.87.14.5533 PMC54359

[36] Hernandez V.J., Bremer H. Characterization of RNA and DNA synthesis in Escherichia coli strains devoid of ppGpp. J. Biol. Chem. 1993; 268:10851–10862. 7684368

[37] Hernandez V.J., Bremer H. Guanosine tetraphosphate (ppGpp) dependence of the growth rate control of rrnB P1 promoter activity in Escherichia coli. J. Biol. Chem. 1990; 265:11605–11614. 2114400

[38] Soupene E., van Heeswijk W.C., Plumbridge J., Stewart V., Bertenthal D., Lee H., Prasad G., Paliy O., Charernnoppakul P., Kustu S. Physiological studies of Escherichia coli strain MG1655: growth defects and apparent cross-regulation of gene expression. J. Bacteriol. 2003; 185:5611–5626. 12949114 10.1128/JB.185.18.5611-5626.2003 PMC193769

[39] Lyons E., Freeling M., Kustu S., Inwood W. Using genomic sequencing for classical genetics in E. coli K12. PLoS One. 2011; 6:e16717. 21364914 10.1371/journal.pone.0016717 PMC3045373

[40] Sun D., Lee G., Lee J.H., Kim H.Y., Rhee H.W., Park S.Y., Kim K.J., Kim Y., Kim B.Y., Hong J.I. et al. A metazoan ortholog of SpoT hydrolyzes ppGpp and functions in starvation responses. Nat. Struct. Mol. Biol. 2010; 17:1188–1194. 20818390 10.1038/nsmb.1906

[41] Mao X.J., Huo Y.X., Buck M., Kolb A., Wang Y.P. Interplay between CRP-cAMP and PII-Ntr systems forms novel regulatory network between carbon metabolism and nitrogen assimilation in Escherichia coli. Nucleic Acids Res. 2007; 35:1432–1440. 17284458 10.1093/nar/gkl1142 PMC1865078

[42] Cayley S., Lewis B.A., Guttman H.J., Record M.T. Jr. Characterization of the cytoplasm of Escherichia coli K-12 as a function of external osmolarity. Implications for protein-DNA interactions in vivo. J. Mol. Biol. 1991; 222:281–300. 1960728 10.1016/0022-2836(91)90212-o

[43] Iyer S., Le D., Park B.R., Kim M. Distinct mechanisms coordinate transcription and translation under carbon and nitrogen starvation in Escherichia coli. Nat. Microbiol. 2018; 3:741. 29760462 10.1038/s41564-018-0161-3

[44] Zhu M., Dai X., Guo W., Ge Z., Yang M., Wang H., Wang Y.P. Manipulating the bacterial cell cycle and cell size by titrating the expression of ribonucleotide reductase. mBio. 2017; 8:e01741-17. 29138305 10.1128/mBio.01741-17 PMC5686538

[45] Ihara Y., Ohta H., Masuda S. A highly sensitive quantification method for the accumulation of alarmone ppGpp in Arabidopsis thaliana using UPLC-ESI-qMS/MS. J. Plant Res. 2015; 128:511–518. 25752614 10.1007/s10265-015-0711-1

[46] Steinchen W., Bange G. The magic dance of the alarmones (p)ppGpp. Mol. Microbiol. 2016; 101:531–544. 27149325 10.1111/mmi.13412

[47] Harinarayanan R., Murphy H., Cashel M. Synthetic growth phenotypes of Escherichia coli lacking ppGpp and transketolase A (tktA) are due to ppGpp-mediated transcriptional regulation of tktB. Mol. Microbiol. 2008; 69:882–894. 18532980 10.1111/j.1365-2958.2008.06317.x PMC2561915

[48] Dai X., Zhu M., Warren M., Balakrishnan R., Okano H., Williamson J.R., Fredrick K., Hwa T. Slowdown of translational elongation in Escherichia coli under hyperosmotic stress. MBio. 2018; 9:e02375-17. 29440576 10.1128/mBio.02375-17 PMC5821080

[49] Ryals J., Little R., Bremer H. Control of rRNA and tRNA syntheses in Escherichia coli by guanosine tetraphosphate. J. Bacteriol. 1982; 151:1261–1268. 6179924 10.1128/jb.151.3.1261-1268.1982 PMC220404

[50] Klumpp S., Hwa T. Bacterial growth: global effects on gene expression, growth feedback and proteome partition. Curr. Opin. Biotechnol. 2014; 28:96–102. 24495512 10.1016/j.copbio.2014.01.001 PMC4111964

[51] Papp-Wallace K.M., Maguire M.E. Manganese transport and the role of manganese in virulence. Annu. Rev. Microbiol. 2006; 60:187–209. 16704341 10.1146/annurev.micro.60.080805.142149

[52] Zhang Y., Zbornikova E., Rejman D., Gerdes K. Novel (p)ppGpp binding and metabolizing proteins of Escherichia coli. MBio. 2018; 9:e02188-17. 29511080 10.1128/mBio.02188-17 PMC5845004

[53] Magnusson L.U., Gummesson B., Joksimovic P., Farewell A., Nystrom T. Identical, independent, and opposing roles of ppGpp and DksA in Escherichia coli. J. Bacteriol. 2007; 189:5193–5202. 17496080 10.1128/JB.00330-07 PMC1951846

[54] Ding C.-K.C., Rose J., Wu J., Sun T., Chen K.-Y., Chen P.-H., Xu E., Tian S., Akinwuntan J., Guan Z Mammalian stringent-like response mediated by the cytosolic NADPH phosphatase MESH1. 2018; bioRxiv doi: 10.1101/325266, 17 May 2018, preprint: not peer reviewed.

[55] Cunningham L., Guest J.R. Transcription and transcript processing in the sdhCDAB-sucABCD operon of Escherichia coli. Microbiology. 1998; 144:2113. 9720032 10.1099/00221287-144-8-2113

[56] Cassan M., Parsot C., Cohen G.N., Patte J.C. Nucleotide sequence of lysC gene encoding the lysine-sensitive aspartokinase III of Escherichia coli K12. Evolutionary pathway leading to three isofunctional enzymes. J. Biol. Chem. 1986; 261:1052–1057. 3003049

[57] Theze J., Saint-Girons I. Threonine locus of Escherichia coli K-12: genetic structure and evidence for an operon. J. Bacteriol. 1974; 118:990–998. 4364333 10.1128/jb.118.3.990-998.1974 PMC246849

[58] Kamarthapu V., Epshtein V., Benjamin B., Proshkin S., Mironov A., Cashel M., Nudler E. ppGpp couples transcription to DNA repair in E. coli. Science. 2016; 352:993–996. 27199428 10.1126/science.aad6945 PMC4917784

[59] Nguyen D., Joshi-Datar A., Lepine F., Bauerle E., Olakanmi O., Beer K., McKay G., Siehnel R., Schafhauser J., Wang Y. et al. Active starvation responses mediate antibiotic tolerance in biofilms and nutrient-limited bacteria. Science. 2011; 334:982–986. 22096200 10.1126/science.1211037 PMC4046891

[60] Pizarro-Cerda J., Tedin K. The bacterial signal molecule, ppGpp, regulates Salmonella virulence gene expression. Mol. Microbiol. 2004; 52:1827–1844. 15186428 10.1111/j.1365-2958.2004.04122.x

