# Supplementary material for: Growth suppression by altered (p)ppGpp levels results from non-optimal resource allocation in Escherichia coli
Source: Nucleic Acids Res. 2019 Mar 27;47(9):4684–93. doi: 10.1093/nar/gkz211 (PMC6511861; doi:10.1093/nar/gkz211)
Supplement: Supplementary Data [file gkz211_supplemental_file.pdf]

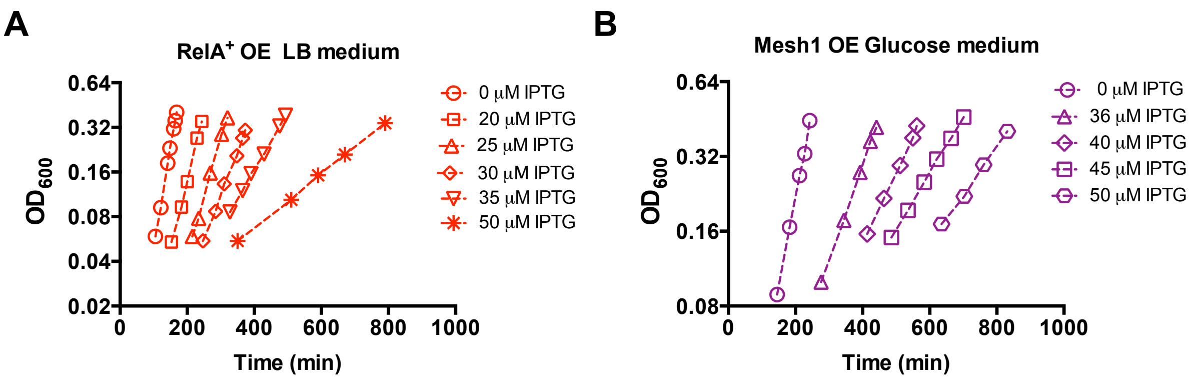

Figure S1 Growth curve of *E. coli* upon RelA$^+$ and Mesh1 overexpression. (A) Growth curve of *E. coli* upon different degrees of RelA$^+$ overexpression (OE) at LB medium. **(B)** Growth curve of *E. coli* cells upon different degrees of Mesh1 overexpression (OE) at glucose minimal medium.

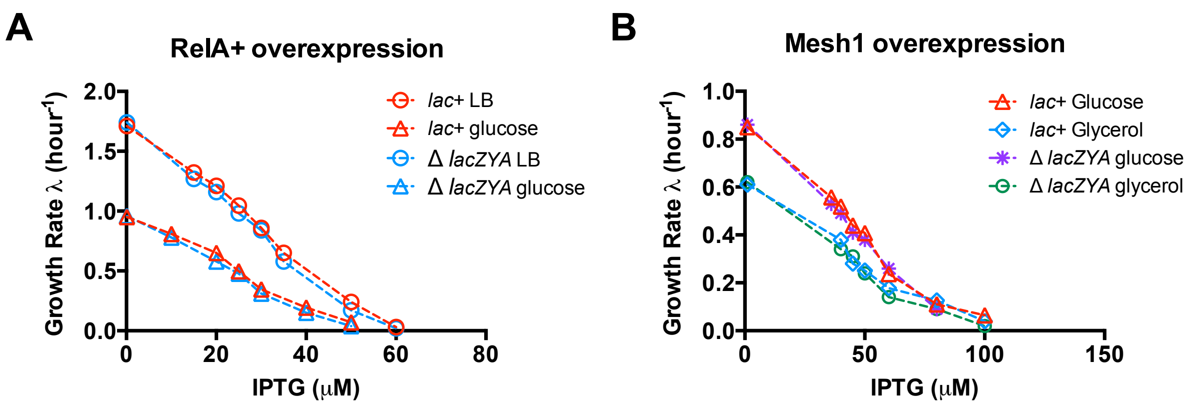

Figure S2 Effect of *lac* operon deletion on the growth rate of *E. coli* upon RelA^{+} and Mesh1 overexpression. (A) Effect of RelA^{+} overexpression (OE) on the growth rate of *E. coli* cells in LB medium and glucose minimal medium. Data of *lac*+ corresponds to the wild type background as shown in Figure 2B. **(B)** Effect of Mesh1 overexpression (OE) on the growth rate of *E. coli* cells in glucose medium and glycerol minimal medium. Data of *lac*+ corresponds to the wild type background as shown in Figure 3C.

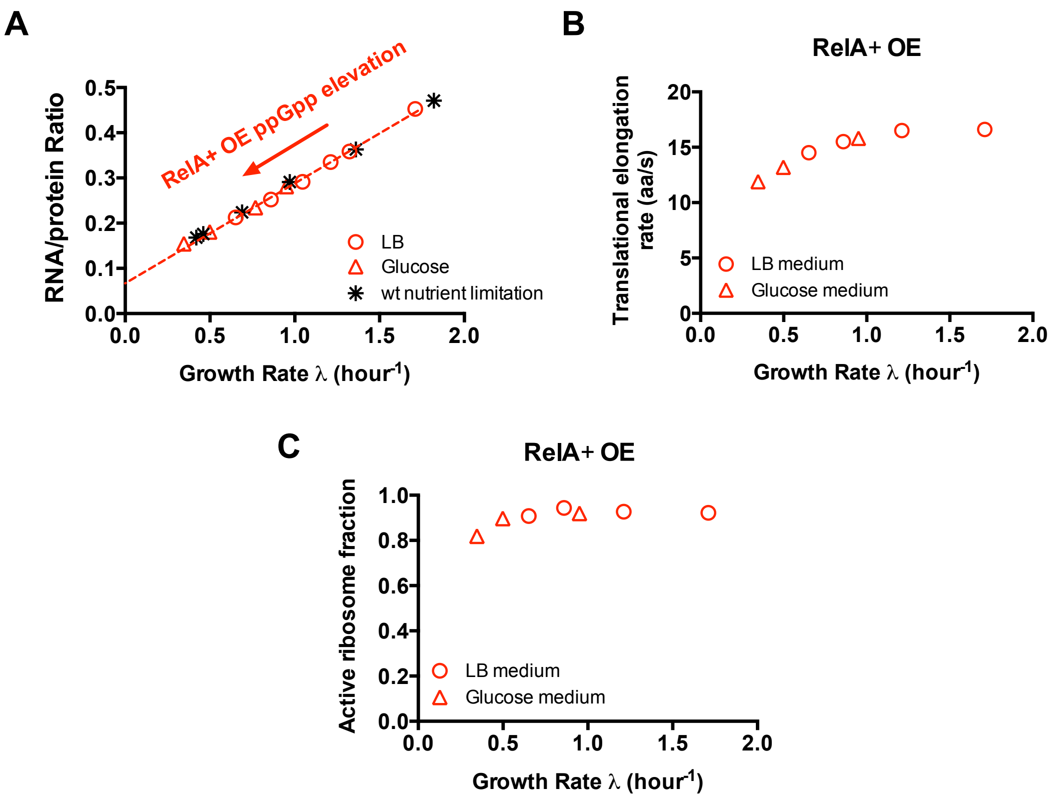

Figure S3 Protein synthesis parameters of *Escherichia coli* under RelA^{+} overexpression. (A) Ribosome content (RNA/protein ratio) plotted against growth rate under different degrees of RelA^{+} protein overexpression. The panel is the same as shown in Figure 2D. **(B)** Translational elongation rate versus growth rate under different degrees of RelA^{+} protein overexpression. **(C)** Active ribosome fraction versus growth rate under different degrees of RelA^{+} protein overexpression. Data points are the average of triplicate determinations. The standard deviations were ~10% (within the size of the symbols). The deduction of active ribosome fraction is based on the mass balance equation shown as shown in Ref. 9 and Ref. 48.

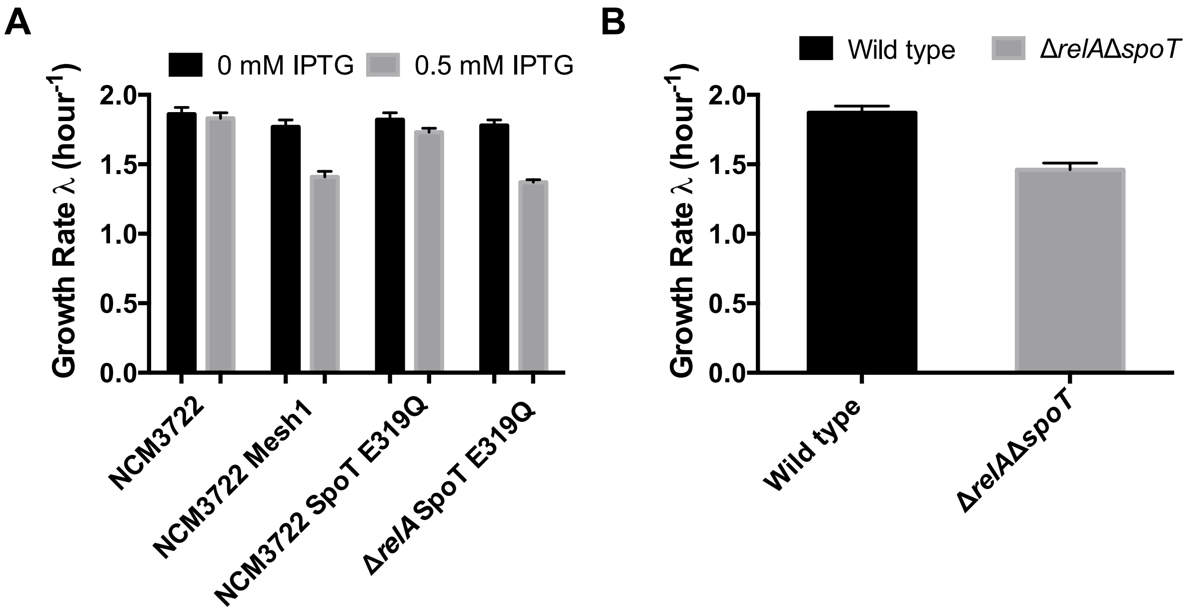

Figure S4 Growth rate of *E. coli* upon ppGpp down-regulation in LB medium. The *E. coli* cells devoid of ppGpp can still grow at a nearly normal growth rate in LB rich medium. **(A)** The growth rate of NCM3722 wild-type cells, NCM3722 Mesh1 cells (*Ptac-mesh1* in the plasmid), NCM3722 SpoT E319Q cells (*Ptac-spoT E319Q* in the plasmid) and Δ*relA* SpoT E319Q cells (*Ptac-spoT E319Q* in the plasmid) in LB medium supplemented with/without 0.5 mM IPTG. **(B)** Growth rate of wild type cells and Δ*relA*Δ*spoT* ppGpp-null cells in LB medium. Data are average of triplicates with standard deviations being within 10%.

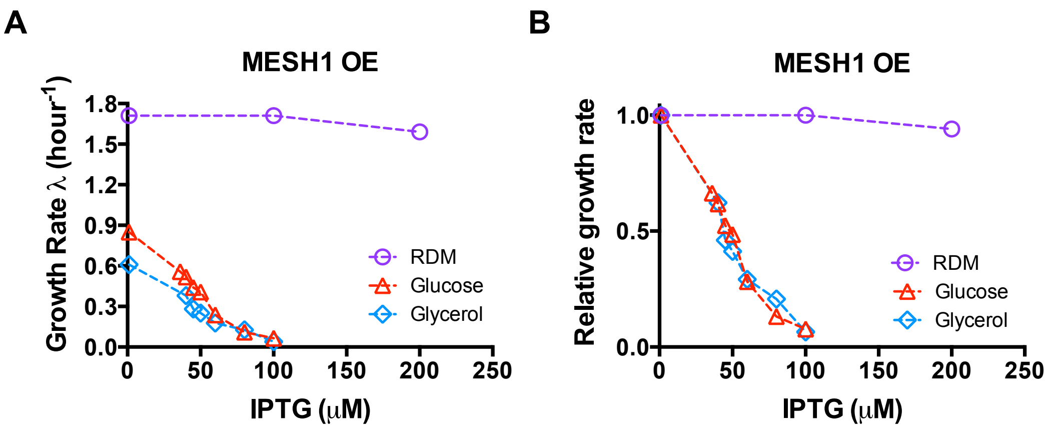

Figure S5 The effect of Mesh1 protein overexpression (OE) on cell growth in rich defined medium. Since LB medium is an undefined medium with complicated compositions, we also repeat the cell growth experiment in rich defined medium (RDM) for Mesh1 protein overexpression. RDM supports almost the same growth rate as LB medium for *E. coli* cells. **(A)** Effect of Mesh1 protein overexpression on the growth rate of *E. coli* in RDM, glucose medium and glycerol medium. **(B)** The relative change of growth rate of *E. coli* upon Mesh1 protein overexpression in each growth condition. The growth rate data of no-IPTG condition in each medium in panel A is set as “1”. Data are average of triplicates with standard deviations being within 10%.

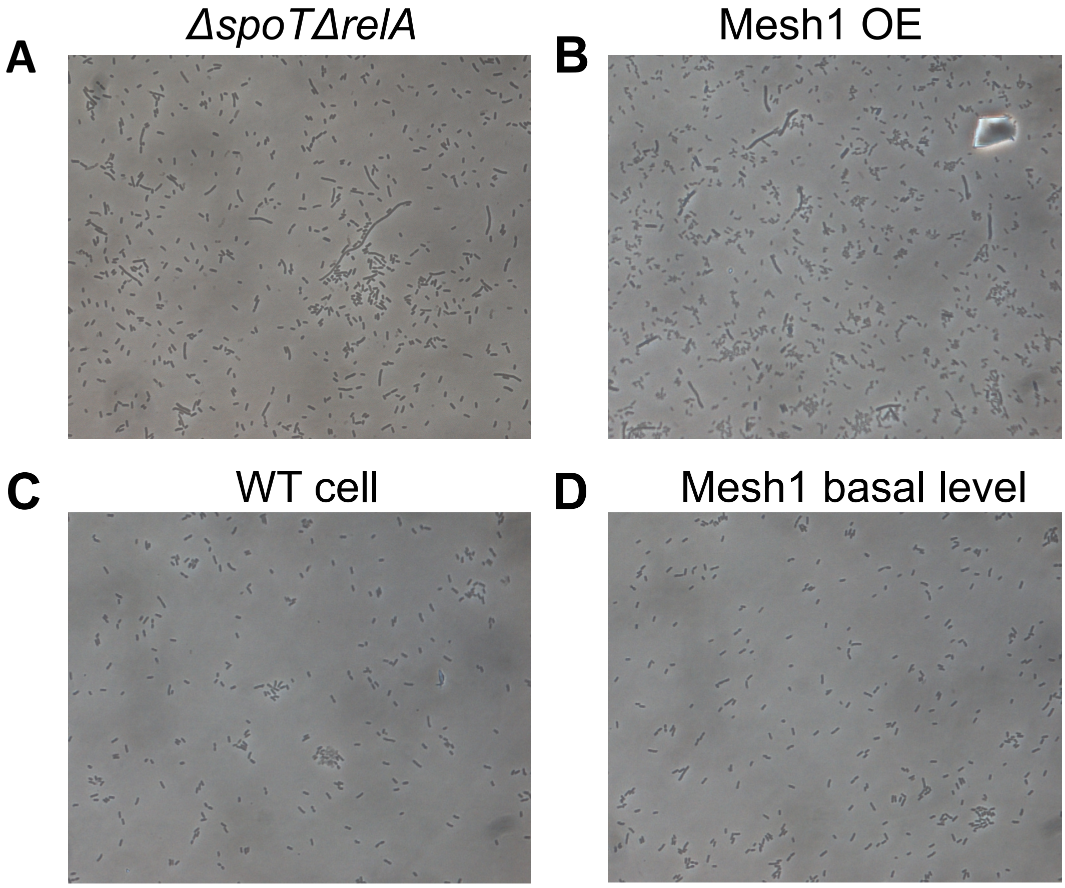

Figure S6 Morphology of cells upon ppGpp down-regulation. The *E. coli* cells were grown in LB medium to stationary phase for cell imaging. Previous studies have found that ppGpp-null strain (Δ*relA*Δ*spoT* double mutant) exhibits filament cell morphology after entering stationary phase (Ref (53)), as also shown in panel A. We found that high degree of Mesh1 overexpression (Mesh1 overexpression strain at 500 μM IPTG) also leads to similar filament cell morphology after entering stationary phase, supporting that ppGpp level has been efficiently down-regulated by Mesh1 overexpression (panel B). In contrast, both wild type cells (panel C) and cells with basal level of Mesh1 protein (panel D) exhibit normal cell morphology during stationary phase.

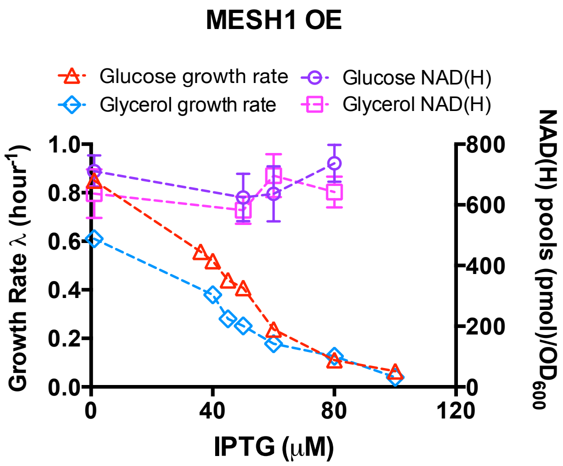

Figure S7 The cellular NAD(H) pool of *E. coli* cells under Mesh1 overexpression. Cells were growing in either glucose minimal medium or glycerol minimal medium. The growth rate data in glucose and glycerol medium is exactly the same as shown in Figure 3C.

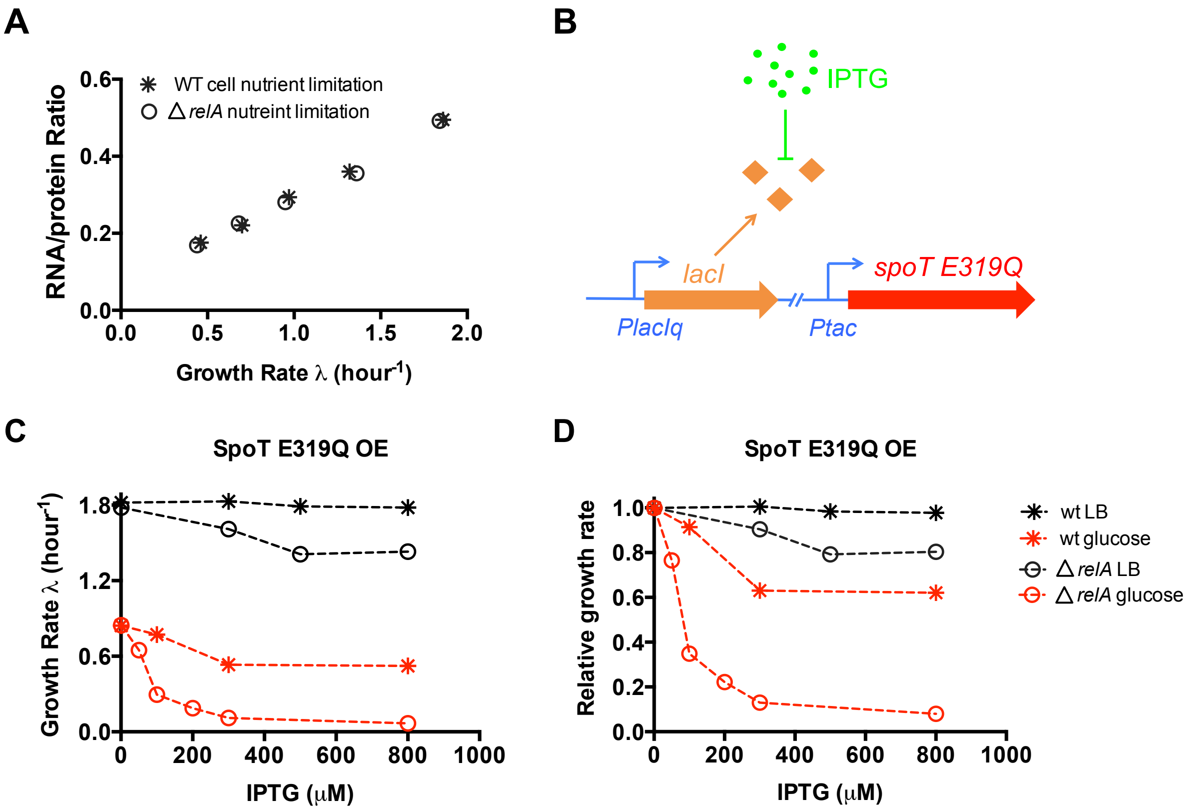

Figure S8 ppGpp down-regulation by the SpoT E319Q protein. (A) Growth rate-dependent ribosome content of wild type cells and Δ*relA* mutant cells under nutrient limitation. Cells were growing in five nutrient conditions including LB medium, glucose casamino acid medium, glucose minimal medium, glycerol minimal medium and acetate minimal medium. **(B)** SpoT E319Q overexpression (OE) based on the inducible *lacIq-Ptac* system. **(C)** Effect of SpoT E319Q over-expression on the growth rate of wild type cells or Δ*relA* mutant cells growing in LB medium or glucose minimal medium. (D) The relative change of growth rate of *E. coli* upon SpoT E319Q overexpression in each growth condition. The growth rate data of no-IPTG condition in each medium in panel C is set as "1". Data are average of triplicates with standard deviations being within 10%.

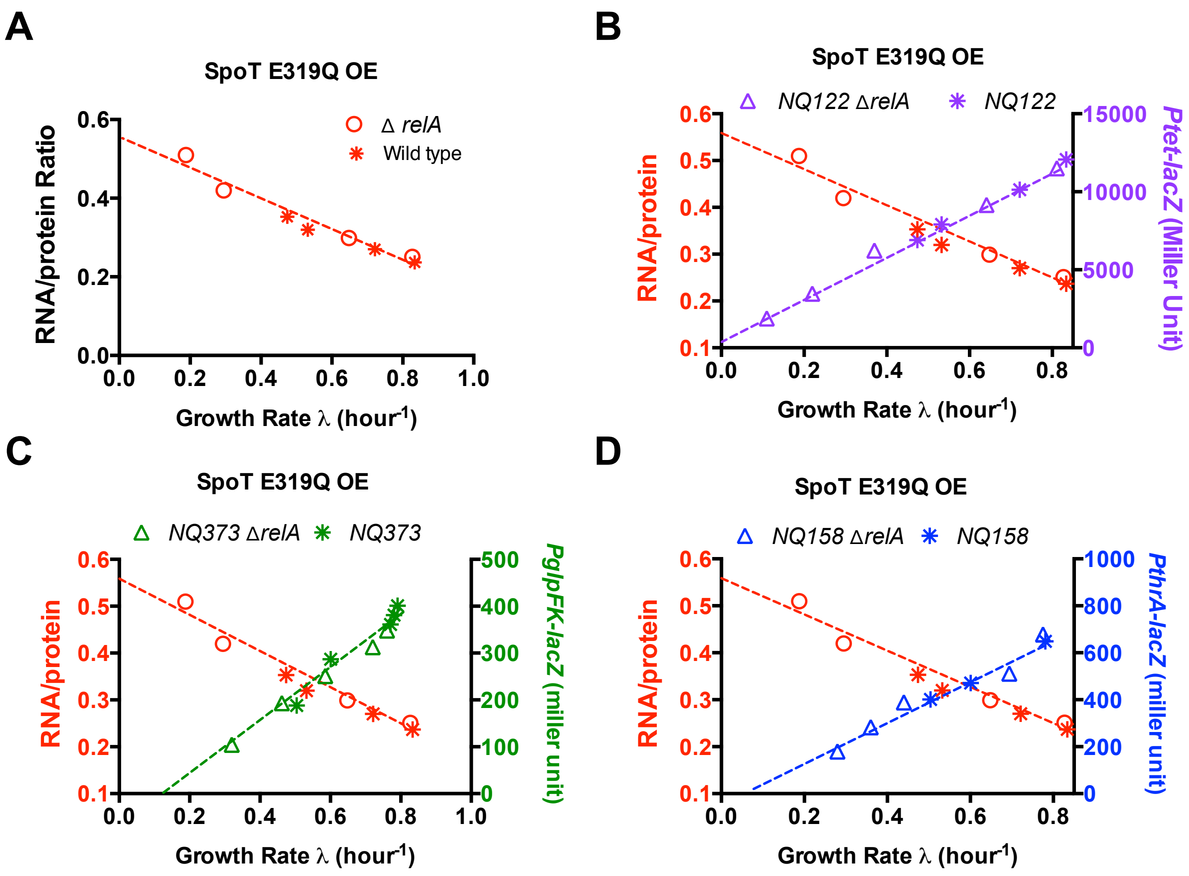

Figure S9 Effect of ppGpp down-regulation (mediated by SpoT E319Q) on the ribosome content and gene expression of *E. coli*. (A) Ribosome content (RNA/protein ratio) plotted against growth rate under different degrees of SpoT E319Q overexpression (OE) at wild type background or Δ*relA* background. **(B)** The constitutive *PLtetO* promoter activity plotted against growth rate under different degrees of SpoT E319Q protein overexpression. **(C)** The catabolic *PglpFK* promoter activity plotted against growth rate under different degrees of SpoT E319Q protein overexpression. **(D)** The anabolic *PthrA* promoter activity plotted against growth rate under different degrees of SpoT E319Q protein overexpression. Data are average of triplicates with standard deviations being within 10%.

Supplementary Text 1 Protein sequence and coding sequence of *mesh1* gene from *Drosophila melanogaster*. The coding sequence has been optimized based on the *E. coli* codon bias.

>Mesh1 protein (179 amino acids)

```
MATYPSAKFMECLQYAAFKHRQQRRKDPQETPYVNHVINVSTILSVEACITDEGVLMAALLH
DVVEDTDASFEDVEKLFGPDVCGLVREVTDDKSLEKQERKRLQIENAAKSSCRAKLIKLADK
LDNLRDLQVNTPTGWTQERRDQYFVWAKKVVDNLRGTNANLELKLDEIFRQRGLL
```

>*mesh1* coding sequence (540 bp)

```
ATGGCTACCTACCCGTCTGCTAAATTCATGGAATGCCTGCAGTACGCTGCTTTCAAACACCG
TCAGCAGCGTCGTAAAGACCCGCAGGAAACCCCGTACGTTAACCACGTTATCAACGTTTCTA
CCATCCTGTCTGTTGAAGCGTGCATCACCGACGAAGGTGTTCTGATGGCTGCTCTGCTGCAC
GACGTTGTTGAAGACACCGACGCTTCTTTCGAAGACGTTGAAAAACTGTTCGGTCCGGACGT
TTGCGGTCTGGTTCGTGAAGTTACCGACGACAAATCTCTGGAAAAACAGGAACGTAAACGTC
TGCAGATCGAAAACGCTGCTAAATCTTCTTGCCGTGCTAAACTGATCAAACTGGCTGACAAA
CTGGACAACCTGCGTGACCTGCAGGTTAACACCCCGACCGGTTGGACCCAGGAACGTCGTGA
CCAGTACTTCGTTTGGGCTAAAAAAGTTGTTGACAACCTGCGTGGTACTAACGCTAACCTGG
AACTGAAACTGGACGAAATCTTCCGTCAGCGTGGTCTGCTGTAA
```
